# *Talaromyces marneffei* Infection: Virulence, Intracellular Lifestyle and Host Defense Mechanisms

**DOI:** 10.3390/jof8020200

**Published:** 2022-02-19

**Authors:** Kritsada Pruksaphon, Joshua D. Nosanchuk, Kavi Ratanabanangkoon, Sirida Youngchim

**Affiliations:** 1 Department of Microbiology, Faculty of Medicine, Chiang Mai University, Chiang Mai 50200, Thailand; kpruksaphon@gmail.com; 2 Department of Medicine, Division of Infectious Diseases, Albert Einstein College of Medicine, Bronx, NY 10467, USA; josh.nosanchuk@einsteinmed.org; 3 Department of Microbiology and Immunology, Albert Einstein College of Medicine, Bronx, NY 10467, USA; 4 Department of Microbiology, Faculty of Science, Mahidol University, Bangkok 10400, Thailand; kavi.rtn@mahidol.ac.th

**Keywords:** *Talaromyces marneffei*, virulence factors, macrophages, immune response, immune escape, innate immunity, acquired immunity

## Abstract

Talaromycosis (Penicilliosis) is an opportunistic mycosis caused by the thermally dimorphic fungus *Talaromyces* (*Penicillium*) *marneffei*. Similar to other major causes of systemic mycoses, the extent of disease and outcomes are the results of complex interactions between this opportunistic human pathogen and a host’s immune response. This review will highlight the current knowledge regarding the dynamic interaction between *T. marneffei* and mammalian hosts, particularly highlighting important aspects of virulence factors, intracellular lifestyle and the mechanisms of immune defense as well as the strategies of the pathogen for manipulating and evading host immune cells.

## 1. Introduction

Among the hundreds of *Talaromyces* species, *Talaromyces* (*Penicillium*) *marneffei* is the only thermally dimorphic species known to be pathogenic to mammals, including humans. *T. marneffei* is a primary lung pathogen that disseminates to other internal organs by lymphatic or hematogenous mechanisms [1]. It causes disseminated disease in both immunocompetent and immunocompromised individuals, though it is most prevalent in patients with HIV/AIDS as well as patients with functional impairments of cellular immunity, particularly defects in CD4 T cell activity. *T. marneffei* grows as a saprophytic mold in the environment, but undergoes phase transition to a pathogenic yeast-like cell at mammalian physiologic temperatures. Dr. Gabriel Segretain is credited with identifying the fungus and he named it in honor of Dr. Hubert Marneffe, the director of the Pasteur Institute of Indochina [2]. Dr. Segretain was the first to note laboratory acquisition of the infection after accidentally inoculated to his finger, which caused axillary lymphadenopathy that resolved spontaneously [3]. Seventeen years later, the first naturally occurring human infection was reported in a 61-year-old American missionary with Hodgkin’s lymphoma who had traveled in Southeast Asia [4].

The first case reports from an endemic area were published in 1984, describing five patients in northern Thailand [5]. In 1985, eight Chinese cases were reported from southern China and Hong Kong. The first case of *T. marneffei* infection in an HIV-infected patient of Southeast Asia was reported in 1989 from Bangkok, Thailand, and this coincided with the beginning of the HIV epidemic in the region [6]. More recently, *T. marneffei* was described in a dog in Brazil co-infected with canine distemper virus (CDV), which represents the first description of talaromycosis in a dog and also extends the geographical distribution of *T. marneffei* outside the known endemic area [7].

In other mammals, *T. marneffei* was first isolated from bamboo rats (*Rhizomys sinensis*) as well as from other species of bamboo rat distributed in an endemic area of *T. marneffei*. The bamboo rat is recognized as an important natural reservoir host of *T. marneffei* [8,9,10]. Up to 75% of wild bamboo rats in Thailand are infected with this fungus with the lungs having the highest rates of disease (83.3%) followed by the liver (33%), which suggests that inhalation of *T. marneffei* results in the establishment of infection in the wild bamboo rat [11]. Significantly, no clinical symptoms of fungal infection were observed in the infected animals, which implicates the bamboo rat as an ecological niche for *T. marneffei* [12].

The mechanisms for the pathogenesis of talaromycosis are not definitively established. However, fungal morphogenesis appears to be a crucial virulence factor in the establishment of infection [13]. Evidence suggests that aerosolized infectious particles (conidia) from environmental disturbances, especially in tropical monsoon seasons, are acquired via inhalation [14,15,16,17]. After inhalation, the sizes of conidia (2 to 3 μm in diameter) allow them to infiltrate deeply into the lung alveoli. Once in the lung, these infectious propagules undergo phase transition into the parasitic yeast form, which are rapidly ingested by lung phagocytes. In healthy individuals, engulfed conidia are, for example, killed by host macrophages through the production of oxidative burst as well as the action of lysosomal enzymes. However, as with other pathogenic fungi such as *Histoplasma*, *T. marneffei* can survive and replicate inside the phagosomal compartment of macrophages [18,19,20,21]. Hence, *T. marneffei* is classified as a facultative intracellular pathogen as it is found inside macrophages and tissue histiocytes in talaromycosis patients [14,22].

The ability to transition from an environmental mold to a yeast form and resist to killing by host phagocyte is recognized as an important virulence mechanism of dimorphic pathogenic fungi [23] as the switch is challenging to host innate and acquired immune defenses. In particular, the pre-existing impairment of cell-mediated immune responses that occur, for example, in patients with AIDS results in severely reduced fungicidal activity that diminishes the capacity of host phagocytes to eradicate this pathogen. In this review, we summarize the current knowledge regarding the dynamic interaction between *T. marneffei* and the host, particularly in the context of virulence factors and mechanisms of host immune defenses.

## 2. Establishment of *T. marneffei* Infection

To establish the infection, pathogenic fungi including *T. marneffei* require to (**I**) adhere to and colonize host tissue, (**II**) multiply, (**III**) escape or destroy host defense system and (**IV**) damage the host tissue [24]. The respiratory portal of *T. marneffei* conidia entry is similar to that resulting in infections caused by another dimorphic fungus [25]. The roles of glyceraldehyde-3-phosphate dehydrogenase (GAPDH) in mediating the adhesion of *T. marneffei* conidia to pneumocytes and human extracellular matrix proteins (ECM) have been reported [26]. Moreover, *T. marneffei* conidia adhere to host ECMs, fibronectin and laminin, via an N-acetylneuraminic acid (a compound rich in terminal sialic acid residues) dependent process [27,28]. Conidia are also able to bind host glycosaminoglycans (GAGs) such as heparin and chondroitin sulfate B [29]. These ECMs are exposed in the lung upon tissue damage and facilitate the attachment of conidia to the bronchioalveolar epithelium. Nevertheless, these interactions have yet to be proven in in vivo *T. marneffei* animal models.

**Figure 1:**
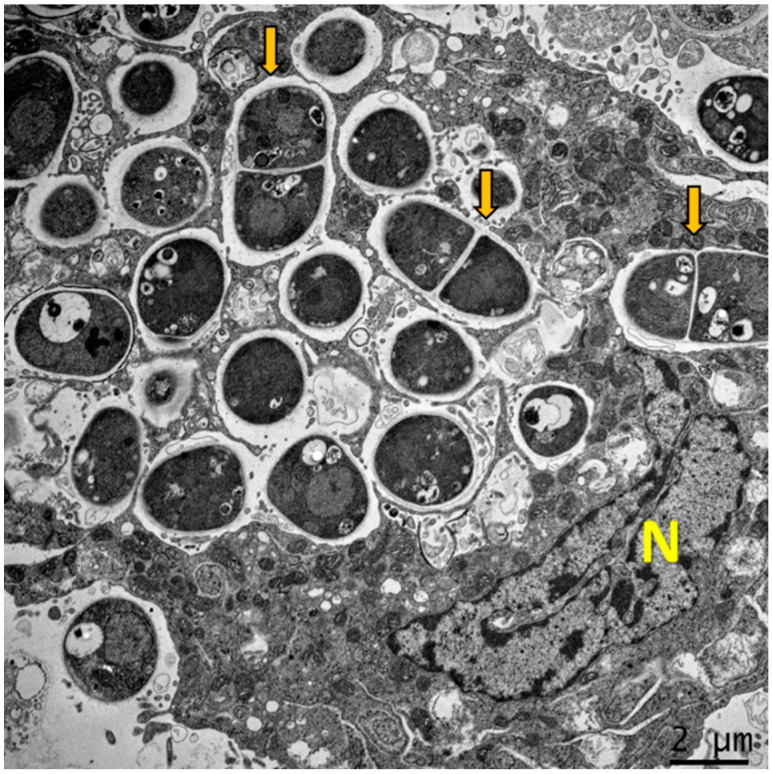
Dimorphism of *T. marneffei* inside macrophage. The major virulence factor of *T. marneffei* is dimorphic switching. Transmission electron microscopy (TEM) of a THP-1 human macrophage infected with *T. marneffei* demonstrates that *T. marneffei* conidia undergo morphogenesis to fission yeast (depict by yellow arrows) inside the phagosome by 24 h after internalization. The photograph was taken under 3000× magnification with a JEM-2200FS (Japan) microscope. N represents the THP-1 nucleus.

After conidial attachment, phagocytic cells are the first line of defense against *T. marneffei*. Conidia are phagocytized by resident alveolar macrophage as well as polymorphonuclear neutrophils (PMNs) [30,31]. In macrophage, they rapidly undergo morphogenic transition to the yeast form and multiply intracellularly. In vitro studies with the murine macrophage cell line (J774A.1) and THP-1 human macrophage cell lines demonstrate that conidia replicate intracellularly by binary fission and express yeast phase specific antigen within 24 h of phagocytosis [20,32] (Figure 1). Subsequently, rather than being eliminated by the natural killing mechanism of the macrophage, *T. marneffei* are able to subvert the natural killing mechanisms of macrophages to survive and replicate inside the phagosome, persisting though lysosome fusion, and finally, escaping into the cytoplasmic environment [22,33].

After establishing infection inside alveolar phagocytes, *T.* *marneffei* (predominantly in the parasitic yeast cell form) can readily spread in a Trojan horse manner within these host cells throughout the host resulting in systemic infection [14,34]. The reticuloendothelial system (RES) associated to internal organs such as lung, liver, lymph nodes, spleen, and bone marrow are commonly involved in disseminated talaromycosis [14]. Instead of acutely causing disease, *T. marneffei* can also become latent with subsequent reactivation, which is clearly evidenced by patients developing talaromycosis years after spending time in an endemic area [35,36,37,38]. The progression and severity of the disease seems to depend on the robustness of the host’s immune status especially in individuals with a low level of CD4 T helper lymphocytes [6,39] or in the presence of neutralizing anti-IFN-γ autoantibodies [40,41,42]. Talaromycosis is frequently observed in advanced stages of HIV infection with CD4 counts below 100 cells/µL and even more frequently when CD4 counts are less than 50 cells/µL [6,39]. Central Nervous System (CNS) infections were historically uncommon, but the incidence has gradually increased in both AIDS and non-AIDS associated talaromycosis [43,44]. Among 677 patients with HIV-associated talaromycosis in Vietnam, 21 (3%) had CNS disease [43,45]. However, the basic mechanisms of *T. marneffei* crossing the human blood–brain barrier is still lacking [44].

## 3. Dimorphism and Intracellular Lifestyle of *T. marneffei*

Unlike various pathogenic bacteria that produced highly potent exotoxins to destroy host tissue, dimorphic pathogenic fungi do not directly produce exotoxin to facilitate the invasion of host tissue [24]. Other major pathogenic fungi such as *Aspergillus* spp., *Candida* spp., and *Cryptococcus* spp. produce diverse virulence factors. For example, *Cryptococcus* spp. secretes hydrolytic enzymes and exotoxins as well as form a unique polysaccharide capsule [46,47]. Less is known regarding virulence factors in *T. marneffei*. For dimorphic fungi, a virulence factor is functionally defined as a factor that affects the survival and growth of the organism in its mammalian host, but is not required for parasitic phase growth in vitro [48]. Indeed, the pathogenesis of *T. marneffei* involves many virulent factors that provide fungal survival and persistence inside the host. Thermal tolerance for survival at temperatures at and above 37 °C is the main prerequisite for dissemination to other host internal organs [14]. Currently, the advantages of modern techniques in molecular genetic based on “Molecular Koch’s Postulates” criteria have been developed to investigate this aspect [49]. A wide range of biological, biochemical, and molecular biological approaches have been used to identify genes and gene products (e.g., phenotypic expression). Many of these gene candidates have been identified by close examination of the characteristics of infection or by expression studies to catalog parasitic (yeast) phase-specific genes [48]. Several genes encoding phase transition and potential virulence factors have been identified to demonstrate the nature of *T. marneffei* virulence determinants [50,51,52,53,54].

Indeed, in dimorphic fungal pathogens such as *Coccidioides*, *Paracoccidioides*, *Histoplasma*, *Blastomyces*, *Sporothrix*, *Emmonsia*, *Emergomyces*, and *T. marneffei*, dimorphic switching from saprophytic mycelium to parasitic phase (e.g., yeasts, spherules, and adiaspores) has been linked to virulence [55,56]. Transition to the parasitic phase offers protection against destruction by phagocytes. Moreover, some fungi have adapted to the stress environment in the phagolysosome or can subvert monocytic cells to enable fungal persistence and proliferation [34,57]. In vitro studies in the context of physico-chemical properties (temperature, pH and saltiness) for controlling phase transitions in *T. marneffei* have been performed. The studies found that optimal temperature for mold growth ranged from 17 to 28 °C. The temperature capable of supporting yeast transition began at 32 °C, but morphogenesis optimally occurred at 37 °C. Also, 40 °C and above inhibits fungal growth. There is a broad range of pH (4.0–10.1) for supporting optimal growth at 37 °C. In addition, NaCl concentrations higher than 4% abrogated hyphal to yeast conversion [58].

Several molecular genetic studies have focused on the genetic element influencing the dimorphic switching of *T. marneffei* [50,52]. Under culture conditions, asexual dormant *T. marneffei* conidia germinate to produce hyphae at 25 to 28 °C. The conidia swell isotropically within the first 6 hr and undergo polarized growth to germinate in the short filament. At 37 °C, the conidia also swell isotropically prior to initiating polarized growth for germination, but the germlings subsequently generate hyphal-like structures that are often highly branched and these develop arthroconidia within 48 h. Eventually, the arthroconidia are liberated, then transform into uninucleate yeast cell arthroconidia that divided by fission [59]. The *abaA* gene is a master regulator of asexual development in filamentous fungi, especially in the aspect of conidiophore development [60]. Borneman et al. (2000) showed that a *T. marneffei abaA* deletion mutant displayed aberrant mold to yeast conversion and arthroconidial filamentations at 25 °C. Likewise, at 37 °C the yeast cell failed to switch correctly from multinucleate filamentous to uninucleate yeast cells and multiple nuclei were observed within either arthroconidium or yeast cells [61].

From antigenic expression studies, dimorphism inside in vitro host macrophages at 37 °C is different from that achieved in artificial culture medium alone [20]. *T. marneffei* infected macrophages contain the unicellular yeast cells which proliferate within these innate immune cells. Within phagocytes, *T. marneffei* conidia directly convert to fission yeast cells in a process called “conidia to unicellular yeast morphogenesis” [59]. Therefore, the genetic elements involved in yeast transition inside host macrophages have been a focus of interest. The hybrid histidine kinase *slnA* and *drkA* genes are essential for dimorphism inside host macrophages at 37 °C. Within 24 h post infection, many fission yeast cells of *T. marneffei* wild type can be observed within conidia infected macrophages. In contrast, infection with *slnA* mutant revealed predominately ungerminated conidia whereas macrophages infected with *drkA* mutant contained mostly septate germlings and a few yeast cells [62]. These findings indicate that *slnA* and *drkA* genes influence germination inside macrophage environments.

There are additional roles for other known *T. marneffei* genes in yeast transition. The yeast cells produced at 37 °C by *rasA* or *cflA* mutants exhibit unusual morphologies that appeared swollen, branched, and missharpened [63,64]. The p21 activated kinase *pakA* and *pakB* that works downstream of RhoGTPase are also involved in conidial germination and correct yeast morphogenesis at 37 °C. The *pakA* mutant remains predominately ungerminated conidia in infected macrophage [65]. However, the deletion of both *pakA* and *pakB* strongly affects the production of yeast cells inside macrophages in vivo, but has no effect in vitro at 37 °C [66]. Recently, a novel Dbl homology/BAR domain protein, MsgA, has been characterized. This gene is upregulated during murine macrophage infection, and deletion results in an atypical yeast morphology during replication inside macrophages. Thus, MsgA plays an essential role in the morphogenesis of yeast cells transition inside host macrophages [21]. Altogether, the genetic controlling of phase transition inside host macrophages at 37 °C is unique when compared to in vitro artificial medium at 37 °C. These data suggested that the conversion of conidia to unicellular yeast morphogenesis program might be triggered by acidic pH, nitrogen source and other certain factors within the cytoplasm of host macrophages [59].

Regarding the intracellular lifestyle of *T. marneffei*, this fungus is likely to have evolved several mechanisms of survival under oxidative stress (or respiratory burst) within the macrophage. Oxidative stress is one of the native defenses produced by phagocytes to kill several intracellular parasitic microorganisms. The phagocytes play a crucial role in eliminating fungal pathogens by producing reactive oxygen or nitrogen species, including superoxide radical anion (O_2_^−^), hydrogen peroxide (H_2_O_2_), hydroxyl radicals (HO•) and nitric oxide (NO) [67]. The reactive oxygen species (ROS) can damage pathogens by altering or inactivating proteins, membrane, nucleic acid, and they have potent immunoregulatory effects on the host immune system that affect the efficacy of the host response [68]. The temporal changes of pH within the phagocytic vacuole may directly reduce fungal growth, or it may inhibit pH dependent yeast virulence factors, such as acid phosphatase activity. Youngchim et al. (1999) investigated an expression of acid phosphatase by *T. marneffei*. When the fungus produces acid phosphatase, it concomitantly might lead to a decrease in host cell intracellular pH and inhibit phagocyte respiratory burst, resulting in enhanced fungal survival [69]. A subsequent study of antimalarial activity of the chloroquine derivative “4-aminoquinoline” against *T. marneffei* in a macrophage cell line infection model showed that 4-aminoquinoline changes macrophage intracellular pH and fungal growth is inhibited [70].

*T. marneffei* produces potent enzymes that specifically detoxify ROS molecules inside host macrophage environments. For example, catalase-peroxidase is a powerful reducing agent that metabolizes H_2_O_2_ to water and oxygen. This enzyme is an established virulence factor in *Mycobacterium tuberculosis* and *Aspergillus fumigatus* [71,72]. The catalase-peroxidase encoding gene (*CpeA*) in *T. marneffei* is associated with the upregulated expression of *CpeA* transcript both in yeast phase and during macrophage infection [73]. Pongpom et al. (2013) showed that *CpeA* controls fungal tolerance to H_2_O_2_ but not to a heat stress response. H_2_O_2_ treatments induced high expression of this gene in both mold and yeast phase. It is therefore proposed that the *CpeA* of *T. marneffei* is utilized to protect conidia and yeast cells from oxidative stress in the host macrophage environment [74]. In addition to catalase-peroxidase, superoxide dismutase (SOD) is an enzyme that alternately catalyzes the dismutation of the superoxide radical (O_2_^−^) into either ordinary molecular oxygen (O_2_) or hydrogen peroxide (H_2_O_2_). *T. marneffei* has been shown to survive and replicate as yeast inside the macrophage phagosome. Thirach et al. (2007) investigated the fungal SOD encoding gene (*sodA*). In *T. marneffei,* the putative SodA peptide consists of 154 amino acids with shared identity to fungal Cu, Zn- SOD. The results suggest that *sodA* might play a role in stress response and in the adaptation of *T. marneffei* inside the macrophage [75].

*T. marneffei* yeast cells must adapt to the stress of glucose starvation while also attempting to exploit alternate carbon sources inside the host-cell environment, since glucose is either limited or absent in the phagosomal environment. Macrophage phagosomes contain a complex variety of alternate carbon sources, including amino acids, carboxylic acids and fatty acids [76]. *T. marneffei* assimilates these alternate carbon sources for energy production in order to survive in this internal macrophage environment [22]. During *T. marneffei* infection, the glyoxylate cycle is induced and *acuE* and *acuD* genes are involved in this process. The *T. marneffei acuD* gene generates isocitrate lyase, the pivotal enzyme in glyoxylate cycle, and gene activity is regulated during the pathogenic yeast phase as *acuD* was highly expressed after macrophage internalization [77]. *acuE* encodes malate synthase, and *T. marneffei acuE* is controlled in a manner similar to *acuD* as that responds to both temperature and acetate induction [78]. Likely most other pathogenic fungi, the availability of iron is crucial to the survival of *T. marneffei* in the human host and the fungus acquires this nutrient using several strategies to uptake host cell iron [79]. The studies by Taramelli et al. (2000) showed that the antifungal activity of macrophages is markedly suppressed in the presence of iron overload and that iron chelators inhibit the extracellular growth of *T. marneffei* [80]. Subsequently, Pongpom et al. (2020) demonstrated the transcription factors responsible for gluconeogenesis and iron acquisition, “AcuM and AcuK,” which play important roles in fungal morphogenesis and survival, and might be contributed in fungal virulence factors [81]. The attenuation of AcuK in a mutant strain of *T. marneffei* increased their susceptibility to killing by THP-1 human macrophages [82]. These data underscore the importance of the ability of *T. marneffei* to assimilate alternative nourishment under glucose restricted conditions.

Since the pathogenic phase of *T. marneffei* is closely linked with growth at higher temperatures, heat shock proteins (HSPs) are proposed as potential virulence factors. HSPs are a group of chaperoning intracellular proteins produced by eukaryotic cells in response to stressful conditions and they are generally upregulated upon infection to prevent misfolding of damaged proteins [83]. *T. marneffei* HSP70 was first isolated and identified by Kummasook et al. (2007). The results showed that the *hsp70* transcription was upregulated during the mycelium to yeast transition. Upregulation was also observed when mycelial or yeast cells were subjected to a heat stress environment at 39 °C. *T. marneffei* HSP70 may play an important role to prevent the yeast proteins from damage during temperature increase [84]. The small HSP30 has also been investigated and transcription is upregulated in the yeast phase grown at 37 °C whereas it is undetectable in the mycelium phase at 25 °C. Thus, HSP30 may play an important role in the heat shock response and in cellular adaptation during infection [85].

Melanin is a dark brown or black pigment of high molecular weight generated by oxidative polymerization of phenolic or indolic compounds. Melanins are produced by a remarkable variety of organisms such as bacteria, fungi, plants and animals. Melanins are involved in virulence of many pathogenic fungi including *H. capsulatum*, *P. brasiliensis*, *C. neoformans*, *A. fumigatus* and *Sporothrix schenckii* [86,87]. Melanin synthesis can promote fungal survival in different environments, synergize their resistance to the immune response in the infected host, and reduce their susceptibility to antifungal agents [87,88]. For example, melanized *C. neoformans* cells are more negatively charged on the cell wall, and this phenomenon interferes with the phagocytotic mechanism [89]. Youngchim et al. (2005) studied melanization of *T. marneffei* in vitro and during infection and found that melanins were generated during infection. In particular, patients with talaromycosis had melanized yeast cells in their skin lesion. Additionally, sera from *T. marneffei* inoculated mice contained IgM and IgG antibodies to fungal melanin. Given the well documented effects of melanin in other pathogenic fungi, *T. marneffei* melanin and melanin-like pigments may have a role in the virulence of this pathogenic microorganism [90]. Subsequently, the gene encoding polyketide synthase (*alb1*) was characterized. These gene clusters were associated with regulating dihydroxynaphthalene (DHN) melanin biosynthesis [91]. Conidia of *T. marneffei alb1* knockdown mutants showed the defects of melanin production and increased the susceptibility to H_2_O_2_. Moreover, the survival rate of BALB/c mice after infection with *alb1* knockdown mutant was higher than for mice infected with the wild-type strain of *T. marneffei* [91,92].

Alongside being involved in melanin generation, p-diphenol dioxygen oxidoreductases or laccases are multi-copper containing oxidoreductase that catalyzes the oxidation of organic and inorganic substances including phenol containing amino acid, methoxy phenol and aromatics amine, with the concomitant four-electron reduction of oxygen to water. The essential properties of fungal laccases have been investigated and were shown to broadly influence fungal development, control phenotype and morphogenesis, detoxify toxins, control pathogenesis in pathogenic fungi and regulate stress response adaptation [93]. Laccases have been associated as contributors to virulence in many fungal pathogens such as *A. fumigatus* and *C. neoformans*. In *C. neoformans*, this enzyme promotes the pathogenicity of *C. neoformans* by catalyzing the formation of melanin precursors. There multiple laccases in *T. marneffei* and the roles of these laccases in virulence and pathogenesis have been characterized. Significantly, a quadruple deletion mutant of *T. marneffei* laccase encoding genes (lac1, lac2, lac3 and arb2) was more susceptible to an oxidative stressor, a cell wall stressor and antifungal agents, including itraconazole, fluconazole and clotrimazole [94]. Subsequently, the mutant *T. marneffei* was also shown to be more susceptible to killing by THP-1 human macrophages compared to infections with wild-type T. marneffei. Moreover, the mutant *T. marneffei* stimulated a significantly higher production of the pro-inflammatory cytokines TNF-α, IL-1β and IL-6 by the THP-1 cells. Altogether, these results demonstrate that *T. marneffei* laccases are involved in resistance to the host immune response [95].

*T. marneffei* genes involved in transcriptional regulation and cellular signal transduction cascade have been studied. The *rfxA* gene is related to nuclear division and binary fission (cytokinesis), and interference with *rfxA* expression caused the defects in cellular division in both conidiation and yeast transition [96]. Furthermore, the impact of *T. marneffei YakA* and *rttA* genes involved in controlling cellular morphogenesis, cell wall integrity and heat stress response have been investigated. YakA plays a crucial role in cell wall integrity and heat stress response at 39 °C. The *rttA* is associated with conidial germination and oxidative stress response. A *Galleria mellonella* infection model at 37 °C demonstrated that *rttA* mutant fails to generate the fission yeast forms in *G. mellonella*, whereas the fission yeasts were found in larvae infected with wild type or complemented strain [97,98]. *T. marneffei* sakA and atfA genes affect the viability of *T. marneffei* conidia under nitrosative and oxidative stresses including adaptation to heat stress at 39–42 °C. Deletion of these genes resulted in the decreased survival rate of conidia inside mouse (J774) and human (THP-1) macrophage cell lines [99,100]. Recently, the gene responsible for the regulation of the methylcitrate cycle (MCD) in *T. marneffei* was examined. Deletion of the MCD gene increased the susceptibility to killing by RAW264.7 mouse macrophage and reduced the mutant strain’s virulence in BALB/c mice [101].

**Figure 2:**
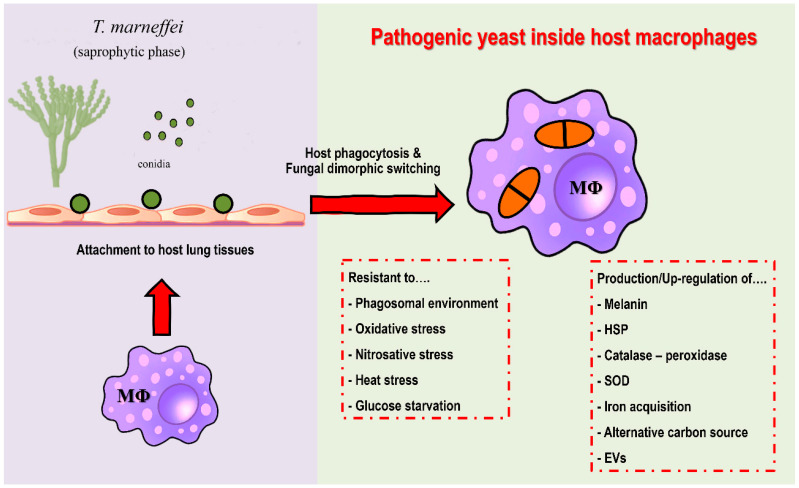
Potential virulence factors of *T***.** *marneffei***.** After attachment to host lung tissue via a host extracellular matrix, host macrophages are the first line of defense against *T. marneffei*. Conidia are phagocytized by alveolar macrophages. After internalization, *T. marneffei* rapidly undergo morphogenic transition to the pathogenic yeast cells and multiply intracellularly. *T. marneffei* then combat various stressors from macrophages, e.g., acidic phagolysosome, reactive oxygen species, reactive nitrogen species, heat stress and glucose deficiency. The fungus is forced to adapt and compensate through virulence factors due to host macrophage pressure, as detailed in the figure. Overall, the traits required for survival and growth in host macrophage environment are considered as potential virulence factors. (Abbreviations: MΦ; macrophages, HSP; heat shock protein, SOD; superoxide dismutase, EVs; extracellular vesicles).

Extracellular vesicles (EVs) are double-layer lipid membrane structures that contain various bioactive components that are released by cells into the extracellular environment. In addition to being released from mammalian cells, bacteria, and diverse other cells, the production and secretion of EVs plays an important role in pathogenic fungal infections [102,103]. Recently, Yang et al. (2021) demonstrated that EVs derived from *T. marneffei* yeast cells are taken up by RAW 264.7 murine macrophage cells and this process increased the expression of surface CD80, CD86, and MHC class II. Moreover, incubation of *T. marneffei* EV with macrophages stimulated the expression levels of ROS, NO, and several inflammatory factors including IL-1β, IL-6, IL-10, and TNF-α. The level of secreted inflammatory factors showed a significant decrease when EVs were destroyed by protease. The proteomic analysis by LC–MS/MS demonstrated *T. marneffei* EV contained highly immunogenic proteins and some bioactive components including heat shock protein, Mp1p mannoprotein, and peroxidase enzyme [104]. The overview diagram of *T. marneffei* potential virulence factor was summarized in Figure 2.

## 4. Host Defense Mechanisms

After encountering *T. marneffei*, the host depends on both innate and acquired immune responses to eradicate the microorganism and combat infection. The mononuclear phagocytes such as macrophage and dendritic cells (DCs) have major roles in the activation of cellular pathways and the production of several cytokines in both pro-inflammatory cytokine (such as IL-1β, TNF-α, IFN-γ) and anti- inflammatory cytokine (IL-10). Recently, an investigation of human genetic polymorphisms was undertaken to predict susceptibility against *T. marneffei* infection. The approach focused on single nucleotide polymorphisms (SNPs) in Toll-like receptor (TLR) genes of Han Chinese with AIDS demonstrated that SNPs within the TLR2, TLR4, and TLR9 genes may contribute to increasing the susceptibility and severity to *T. marneffei* [105]. Moreover, the presence of anti-IFN-γ autoantibodies in adult-onset acquired immunodeficiency (AOID) was significantly associated with HLA-DRB1*16:02 and HLA-DQB1*05:02 alleles in these patients [41].

### 4.1. Innate Immunity

The association between *T. marneffei* infection and immunocompromised status resulting from various factors of acquired immunity impairment strongly suggests that host innate immunity must play a crucial role in controlling *T. marneffei* infection when CD4 T cells are dysfunctional or disabled. Accordingly, the phagocytic cells of the innate immune system including monocytes [106], macrophages [22,33], PMNs [31,34,107], and DCs [108] are involved in combatting *T. marneffei* inside the host.

As mentioned above, macrophages have a central role in the interaction between *T. marneffei* and the host. In addition to human macrophages, the responses of pulmonary macrophages from rabbits and mice to *T. marneffei* conidia have been reported [109,110]. The initial interactions between *T. marneffei* conidia to monocytes/macrophages was demonstrated by Srinoulprasert et al. (2009) who found that engagement of the fungus could be significantly inhibited by monoclonal antibodies against pattern recognition receptors (PRRs), including a Mannose receptor, TLR1, TLR2, TLR4, TLR6, CD14, CD11a, and CD18. Additionally, monocytes co-cultured with *T. marneffei* conidia had increased expression of surface CD40 and CD86 molecules as well as higher TNF-α and IL-1β production [106].

Interestingly, macrophages respond differently to *T. marneffei* conidia and yeast cells. A disparity in the susceptibility of *T. marneffei* conidia and yeast cells to the fungicidal activity of macrophages has been observed in vitro [111], with yeast cells appearing to be more susceptible than conidia to the fungicidal activity of mouse macrophages activated by IFN-γ. In vitro experiments show that exogenous IFN-γ plays a pivotal role in macrophage resistance to *T. marneffei*, and fungicidal activity is mediated by nitric oxide (NO) [32]. Indeed, other in vitro experiments with mouse J774 macrophages have determined a direct relationship between the antifungal activity of IFN-γ stimulated macrophages and NO production [32]. Kudeken et al. (1998) have also demonstrated the antifungal activity of IFN-γ stimulated human macrophage against *T. marneffei* in vitro. These observations indicate that IFN-γ is a potent inducer for killing *T. marneffei* inside macrophages via a NO-dependent process [111].

TNF-α plays an important role in host defense against *T. marneffei*. Using heat-killed yeast, monocyte-derived macrophages readily internalized yeast even in the absence of opzonization and the major receptor(s) recognizing *T. marneffei* included a glycoprotein with N-acetyl-beta-D-glucosaminyl groups. Although *T. marneffei* stimulates the oxidative burst (respiratory burst) of macrophage regardless of whether opsonins are present, TNF-α production is upregulated only in the presence of opsonins. Thus, the ability of un-opsonized *T. marneffei* to parasitize macrophages without stimulating the production of TNF-α might be a critical step for the survival of this intracellular fungus [112].

In addition to macrophages, PMNs have fungicidal activity against *T. marneffei*. Wright’s-stained peripheral blood smears from patients with AIDS-associated *talaromycosis* have revealed large numbers of fission yeast cells inside PMN*s* [30]. Likewise, dysplastic neutrophils appear to increase the risk of talaromycosis [113]. In general, PMNs exert their antifungal effect through two well-known mechanisms. The first is through the actions of ROS, such as H_2_O_2_ and superoxide anion, mediated by enzyme myeloperoxidase, while the second is due to the action of anti-microbial enzymes exocytosed from their granules, such as lysozyme, lactoferrin, acid phosphatase, β-glucuronidase and elastase [114]. In vitro studies have determined that human PMNs can suppress the growth and phase transition of *T. marneffei* yeast cells. Granulocyte-macrophage-colony-stimulating factor (GM-CSF) stimulated PMN*s* demonstrate strong killing activity against T. marneffei yeast forms, but not in conidia. It is speculated that T. marneffei yeast cell killing is executed through exocytosis of PMNs granular enzyme with direct (close) contact between fungus and PMN*s*. Moreover, the killing mechanism of GM-CSF stimulated PMN*s* on *T. marneffei* was not mediated by a superoxide dependent mechanism, because SOD failed to inhibit the fungicidal activity of GM-CSF-stimulated PMNs [31,107].

Recently, the relationship between macrophage and PMN in the pathogenesis of talaromycosis was explored in an ectothermal zebra fish model. In zebra fish, despite the lower incubation temperature (33 °C), the phase transition from conidia to yeast occurs. Additionally, filamentous forms of *T. marneffei* can be observed growing inside PMN whereas yeast forms were predominantly growing within macrophage. This observation implies that the intracellular milieu of those phagocytes may be a determinant of *T. marneffei* morphogenesis in vivo, which can override the influence of temperature. Moreover, this result may also explain the reason why macrophages are the preferred residence for infection and replication of *T. marneffei* as macrophage can serve as a protective shield for the yeast cells to evade PMN mediated destruction by the strong myeloperoxidase activity delivered in PMN granules [34,115].

Although macrophages are considered the key primary effector cells in host resistance against *T. marneffei* [22,33]. However, *T. marneffei* and other pathogenic fungi have developed several strategies to escape host macrophage killing. For example, the attenuation of M1 and/or induction of M2 macrophages polarization are major immune escape tactics [116]. Macrophages are the professional antigen presenting cells (APCs) to prime T cells and manipulate acquired immune responses that promote fungal clearance or accidentally enhance fungal survival [19]. For this reason, macrophage polarization can be classified as either proinflammatory, traditionally activated (M1) or anti-inflammatory, or alternatively activated (M2) macrophages. The expression of certain markers is used to determine the M1/M2 classification [117]. M1 macrophages are typically associated with immunological responses to intracellular infections. Moreover, M1 macrophages are involved in pro-inflammatory responses regulated by T_H_1 signaling. On the other hand, M2 macrophages are involved in anti-inflammatory responses and tissue repair mediated by T_H_2 signaling and are associated with an immunological response to parasitic infections or allergic asthma [118]. In *Candida albicans*, the fungus achieves immune escape via inducing macrophage M2 polarization [119]. In *Cryptococcus neoformans*, the fungus drives monocytes to affiliate an M2 macrophage polarization, which is permissive to fungal proliferation and spreading, in a disseminated model of cryptococcosis [120]. *T. marneffei* stimulated macrophages induce M2 response, mediated by IL-10, in both a BALB/c mouse alveolar macrophage model [121] and a PBMC human macrophage model [122]. Moreover, the inhibiting effect against M1 polarization has also been reported in *T. marneffei* infected THP-1 human macrophages by disturbances CD86 (B7-2) expression [123]. The studies by Wei et al. (2021) demonstrated that SOCS3-STAT6 intracellular signaling components and the TLR9 signaling pathways directly participated in macrophage M2-like polarization and these investigators hypothesized that *T. marneffei* may escape macrophage killing to proliferate inside macrophage by inducing M2-like polarization [124]. The cytokine signaling pathway in *T. marneffei* infected human macrophage have been investigated. Chen et al. (2014) demonstrated that extracellular signal-regulated kinases 1 and 2 (ERK1/2) are essential for TNF-α production, whereas p38 mitogen activated protein kinase is essential for IL-10 production. These findings suggest that ERK1/2 might be essential for the initiation of proinflammatory responses to *T. marneffei*. In contrast, the p38 pathway activation may attenuate host immune response and promote the intracellular survival of *T. marneffei* [125]. Other immune escape strategies in *T. marneffei* include the down regulation of the proinflammatory cytokine IL-6 produced by bronchial epithelial cells [126] and through the expression of yeast cell wall mannoprotein Mp1p, which effectively binds arachidonic acid and suppresses the host pro-inflammatory response [127,128].

DCs are considered the most powerful of the immune phagocytes. In fungal infection, DCs are extremely efficient at processing and presenting fungal antigen to CD4 T cells or alternatively through CD8 T cells (i.e., cross priming or cross presentation) [129]. Unfortunately, there are limited studies to date on the interaction between *T. marneffei* and host DCs. Nakamura et al. (2008) demonstrated that TLR2 and dectin-1 are essential in recognizing *T. marneffei* for the activation of bone marrow-derived DCs (BMDCs) [130]. In a recent study, murine BMDCs recognizing *T. marneffei* yeast cells were found to increase regulatory T cell (Treg) expansion and restrict T_H_17 cell responses by increased the production level of CD80, CD86, IL-6, IL-10 and TGF-β levels in the culture supernatant of T. marneffei-stimulated BMDCs [108]. In the context of pathogenesis, monocyte derived DCs (MDDCs) stimulated with *T. marneffei* enhance HIV-1 trans-infection of primary CD4 T cells [131].

Although cytokine responses are very complex in *T. marneffei* infection and evolve over the course of disease [132], a significant production of pro-inflammatory cytokine (TNF-α, IL-1β and IL-6) in the context of co-cultured between monocytes/macrophages and *T. marneffei* conidia or yeast cells are often reported [20,95,106,112]. The production of IL-1β occurs in response to NLRP3 inflammasome induction by *T. marneffei* yeast cells, which is absent when conidia are used. From in vivo studies, NLRP3 deficient mice have higher fungal loads and increased mortality rates than wild-type mice after systemic *T. marneffei* infection [133]. This study is consistent with our previous report in which the concentrations of IL-1β secreted from infected THP-1 were significantly increased after shifting from conidia to yeast inside THP-1 macrophage [20]. The studies by Dong et al. (2020) reveal that mainly macrophage-derived inflammatory cytokines including TNF-α, IFN-γ, IL-6, IL-12, IL-18, IL-1β, and IL-8 as well as certain chemokines, especially IP-10, play an important role in resistance to *T. marneffei* in patients who suffer from substantial loss of CD4 T cells or other functional impairment of acquired immunity as a result of HIV manifestation. Notably, patients with co-infections of *T. marneffei* and HIV with the most robust proinflammatory cytokine response or “cytokine-storm” had the worst outcomes, underscoring the need for a balanced immunological response [132].

### 4.2. Acquired Immunity

Although depletions of CD4 T lymphocytes are closely associated with an increased risk for talaromycosis, there are relatively few studies specifically investigating the role of T-cell (CD4 and CD8)-mediated immune responses to *T. marneffei*. However, in vivo model studies have determined that T lymphocytes play a protective role against *T. marneffei* infection. Indeed, there is a faster progression of *T. marneffei* infection in athymic nude mice compared to immunologically intact mice [134]. Notably, adoptive T cell transfer from immunocompetent mice infected with *T. marneffei* to infected athymic nude has a profound effect on disease progression as it results in decreased numbers of viable *T. marneffei* cells in the lungs, livers and spleens of the animals receiving adoptive T cell transfer compared to non-treated infected athymic nude mice. These data show that T cell mediated immunity has a central role in host resistance to *T. marneffei* [135].

In talaromycosis patients co-infected with HIV, the histopathologic features depend on the degree of immunological suppression [136]. At lower CD4 T cell counts, talaromycosis tends to be more invasive, and is characterized by an ineffective immune response as indicated by the absence of granulomas and more extensive proliferation of extracellular yeast and intracellular forms within lipid-loaded macrophages (i.e., foam cells). Yeast or yeast-like sausage shaped *T. marneffei* cells are occasionally identified in the peripheral blood by light microscopy of blood smears [136,137]. In patients without advanced HIV or individuals with other immune defects who have normal numbers of CD4 T cells, IFN–γ is pivotal for the host’s resistance to disseminated infection with *T. marneffei*. Notably, high titers of anti-IFN–γ neutralizing autoantibodies in AOID are associated with disseminated non-tuberculous mycobacteria (NTM), histoplasmosis, cryptococcosis, melioidosis, non-typhoidal salmonellosis, and varicella zoster virus infections as well as talaromycosis, which exemplifies the role of IFN –γ in regulating a host’s ability to combat diverse intracellular pathogen [42,138,139,140]. In an experimental animal model, IFN-γ knockout BALB/c mice experienced rapid death after infection with *T. marneffei*, and their spleens and livers showed abundant yeast cells and an absence of granuloma formation. Moreover, T_H_1-polarization of cytokines with increases in IFN- γ and IL-12 was observed in the spleen [141]. These findings are consistent with the fundamental knowledge that a T_H_1 immune response plays a crucial role in host defense to other intracellular pathogenic microorganism such as in infections with mycobacteria [142] and intracellular fungal pathogens, such as *H. capsulatum* and *C. neoformans* [134,143].

In addition to T_H_1, another subset of CD4 T cells is the “T_H_17 and regulatory T cell (Treg)” that play an important role in the dynamic immune balance (or Yin-Yang balance) of protective immunity and autoimmunity as well as immunopathogenesis [144,145]. In *H. capsulatum* infection, increases of T_H_17 cytokines and reductions in the number of Treg are associated with rapid fungal clearance in CCR5 deficient animals [146]. Moreover, in paracoccidioidomycosis, Treg cells promote fungal dissemination whereas depletion of Treg cells promotes T_H_1/T_H_17 protective immunity and prevents fatal disease outcomes from the fungus [147]. Unfortunately, the role of these CD4 T cells in the context of *T. marneffei* infection is not well-defined. Tang et al. 2020 demonstrated that after recognizing *T. marneffei* yeast cells, murine BMDCs increased Treg expansion by upregulating Foxp3 expression and restricting T_H_17 cell responses by downregulating RORγt expression. This phenomenon promotes immunological tolerance and thereby may be harmful to the host defense against *T. marneffei* infection [108]. Overall, the immune deficiencies involving cellular mediated immune responses and CD4 lymphopenia (e.g., T_H_1 defects including problem in IFN- γ/IL-12 production, T_H_17 defect, STAT1 mutation, STAT3 mutation, and CD40 ligand deficiency) are documented to be associated with talaromycosis and therefore are linked to increased susceptibility to *T. marneffei* infection [1].

Due to the immunocompromised status of the majority of individuals with talaromycosis, it is expected that there will be considerable defects of the humoral (antibody mediated) immune responses [148]. Furthermore, antibody responses generally have a restricted role in the clearance of intracellular pathogens [149]. Therefore, the protective role of humoral mediated immunity in *T. marneffei* infection is unclear and, as no major investigations into this aspect of host immunity have been carried out to date, antibody mediated protection has yet to be effectively defined. However, the protective effect of antibodies against cell wall mannoprotein has been reported. Wong et al. (2002) demonstrated the production of murine specific IgM and IgG antibody responses to a highly immunogenic secreted cell wall mannoprotein, Mp1p, using both recombinant protein and DNA immunization strategies [150]. Nevertheless, the effect of these antibodies in disease is unknown.

Indeed, the majority of data regarding humoral mediated immunity to *T. marneffei* has been obtained as a result of serological assays for clinical laboratory diagnosis [151], in which strongly immunoreactive proteins or glycoproteins (e.g., crude cytoplasmic yeast antigen, Mp1p, Mplp6 and HSP30) have been identified as a result of their recognition by serum specific antibodies [85,152,153,154]. Notably, there are low or undetectable levels of antibodies to Mp1p in HIV-infected patients with talaromycosis [148,155]. Moreover, some highly immunogenic *T. marneffei* proteins failed to induce antibody production in immunocompromised patients [152,156].

There is, however, an important link between humoral and cellular immunity that is pertinent to talaromycosis, as protective antibodies are required for robust CD4 T cell-mediated isotype switching and affinity maturation [157]. In the context of immunodeficiency, particularly in the setting of AIDS, perturbations of B cell function prevent the efficient mounting of high-affinity antibody responses against HIV as well as other pathogens [158]. As a consequence, antibody production theoretically could provide initial protection, but without further arming through interactions with CD4 T cells, there are no sustained functional antibodies for prolong protection. Notably, such an antibody production defect has been observed in other AIDS-associated systemic mycosis due to dimorphic fungus including histoplasmosis and blastomycosis [159].

## 5. Conclusions and Future Perspective

**Figure 3:**
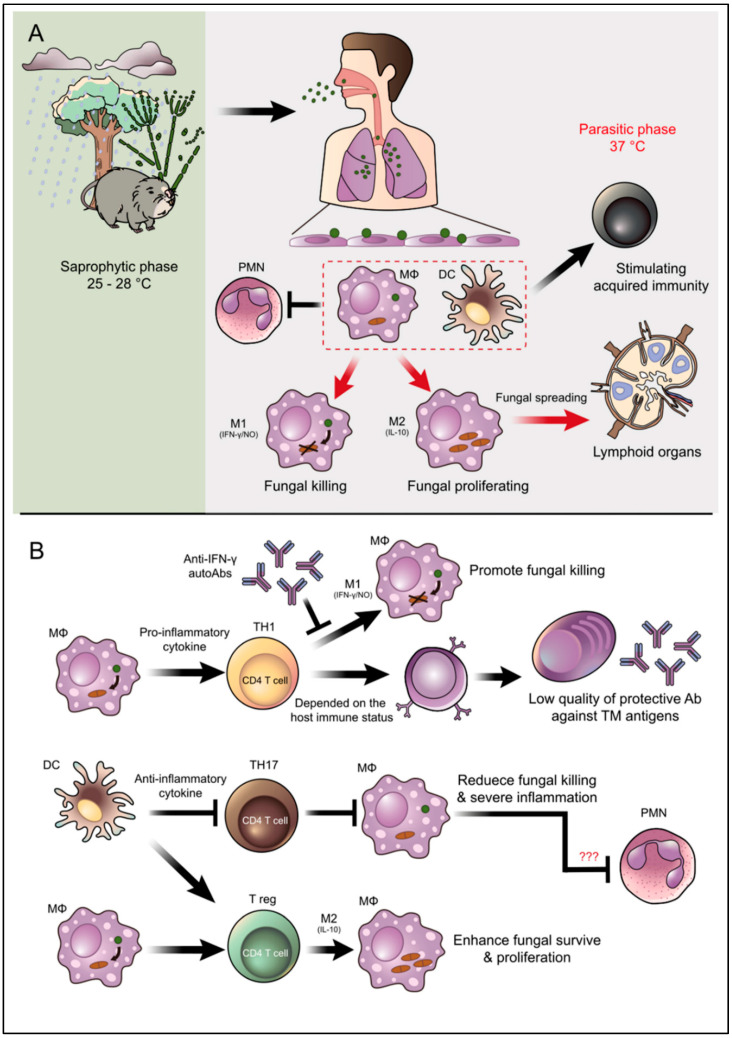
Overview illustration of infection, pathogenesis, and host defense mechanisms of *T. marneffei*. (**A**) *T. marneffei* grows in a saprophytic mold phase in the environment of endemic areas. Bamboo rats are recognized as an important natural reservoir host of *T. marneffei* although the fungus is also soil associated. Infected individuals presumably inhale the aerosolized infectious particle (conidia) after disturbances in the environmental, which is especially common during tropical rainy seasons. *T. marneffei* conidia then travel down to the lung and attach to lung epithelial cells. These infectious propagules are rapidly taken up by pulmonary phagocytes, especially dendritic cells and macrophages, where they undergo phase transition into the parasitic yeast form. Dendritic cells and macrophages are immune cells that effectively link the innate and acquired arms of the immune system. (**B**) The illustration depicts important interactions between *T. marneffei* and these key host effector cells as well as PMNs that regulate disease outcomes. M2 macrophage polarization encourages *T. marneffei* utilities macrophages as habitats, this phenomenal classified as an important factor for fungal immune escape. Due to T_H_17 are importantly in PMNs recruitment, the inhibition of T_H_17 might be related to the suppressive function of PMNs to kill the fungus. However, this occurrence is still just a hypothetical explanation and needs further investigation (indicate by red question marks). The arrow indicates activation/stimulation and the T-bar indicates inhibition.

As reviewed in this paper, researchers in several countries are working to characterize the putative virulence factors of *T. marneffei* and decipher the processes that the fungus utilizes to modify the capacity of the host to combat infection. We highlight that the complexity underlying *T. marneffei*’s ability to establish infection from morphogenesis to intracellular survival. *T. marneffei* is remarkable in its capacity to adapt, survive and manipulate host responses, particularly phagocytic immune cells and especially macrophages. These complex strategies developed by *T. marneffei* are presented in Figure 3.

Although more than six decades have passed since Dr. Segretain discovered *T. marneffei*, there are numerous questions that need to be addressed to robustly understand the biology and pathogenesis of *T. marneffei* [160]. For example, the immunobiology of *T. marneffei*, especially in the context of pathway to survive intracellularly in infected cells (Trojan horse model) [161] and acquired (T cell-mediated) immunity, remain poorly understood. The role of other innate immune cells responsible for *T. marneffei* infection, such as DCs, natural killer cells (NK cells) as well as other innate-like T lymphocytes, are rich areas for exploration. Likewise, the role of other CD4 T cells subset (e.g., T_H_17, Treg, and cytotoxic (CD8) T cells) are urgently needed areas for investigation. Studies on these research areas as well as rigorous explorations of antibodies-based therapy, such as the passive administration of therapeutic monoclonal antibodies [162], prophylactic vaccines to *T. marneffei*, or the utilization of anti-CD19 chimeric antigen receptor-modified T cell (CAR-T-cell) therapies [163] will be important next steps to advance our knowledge and increase our capacity to care for with or at risk for talaromycosis.

## Abbreviations

Treg; regulatory T cell, IFN-γ; interferon gamma, NO; nitric oxide, IL-10; interleukin 10, MΦ; macrophages, PMNs; polymorphonuclear neutrophils, DCs; dendritic cells, T_H_1; T helper 1 lymphocytes, T_H_17; T helper 17 lymphocytes, Treg; regulatory T lymphocytes, Ab or Abs; antibody (or antibodies), TM; *Talaromyces marneffei*).
